# Gas necrosis and sepsis due to recreational ketamine use

**DOI:** 10.1590/S1678-9946202567009

**Published:** 2025-02-07

**Authors:** Alexandre Sacchetti Bezerra, Carla Maria Pasquareli Vazquez, Ana Carolina Troise Guilherme, Ana Beatriz Retamero Rodrigues, Murillo Barbosa Crivillari, Wladimir Queiroz

**Affiliations:** 1 Faculdade de Medicina do ABC, Santo André, São Paulo, Brazil; 2 Hospital Israelita Albert Einstein, São Paulo, São Paulo, Brazil; 3 Instituto de Infectologia Emilio Ribas, São Paulo, São Paulo, Brazil; 4 Faculdade Santa Marcelina, São Paulo, São Paulo, Brazil; 5 Universidade Santo Amaro, São Paulo, São Paulo, Brazil

**Keywords:** Ketamine, Sepsis, Necrosis, Tetanus, Amputation

## Abstract

Although ketamine is an FDA-approved drug, its mechanism of action is not fully understood. Currently, there is an increase in its recreational use, causing irreparable social and physical damage. We report the case of a musician who developed sepsis due to gas necrosis in his arm after using veterinary ketamine purchased via the internet. Despite the amputation recommendation, it was possible to save the arm and preserve motor and sensory function. The scientific community, as well as the police and the government, must ponder the prescription, efficacy and safety of ketamine for medical treatments.

## INTRODUCTION

Ketamine is a racemic mixture of two enantiomers, R-ketamine (arketamine) and S-ketamine (esketamine)^1-4^. It is a controlled substance approved by the United States’ Federal Drug Administration (FDA) as an intramuscular or intravenous injection for induction and maintenance of general anesthesia, indicated for short-term surgeries that do not require muscle relaxation^4,5^. Esketamine has been approved as a nasal spray for depressive symptoms and treatment-resistant depression in adults^6,7^. In the literature, there is no report of sepsis being caused by deltoid necrosis after intramuscular ketamine use.

## CASE REPORT

**Figure 1:**
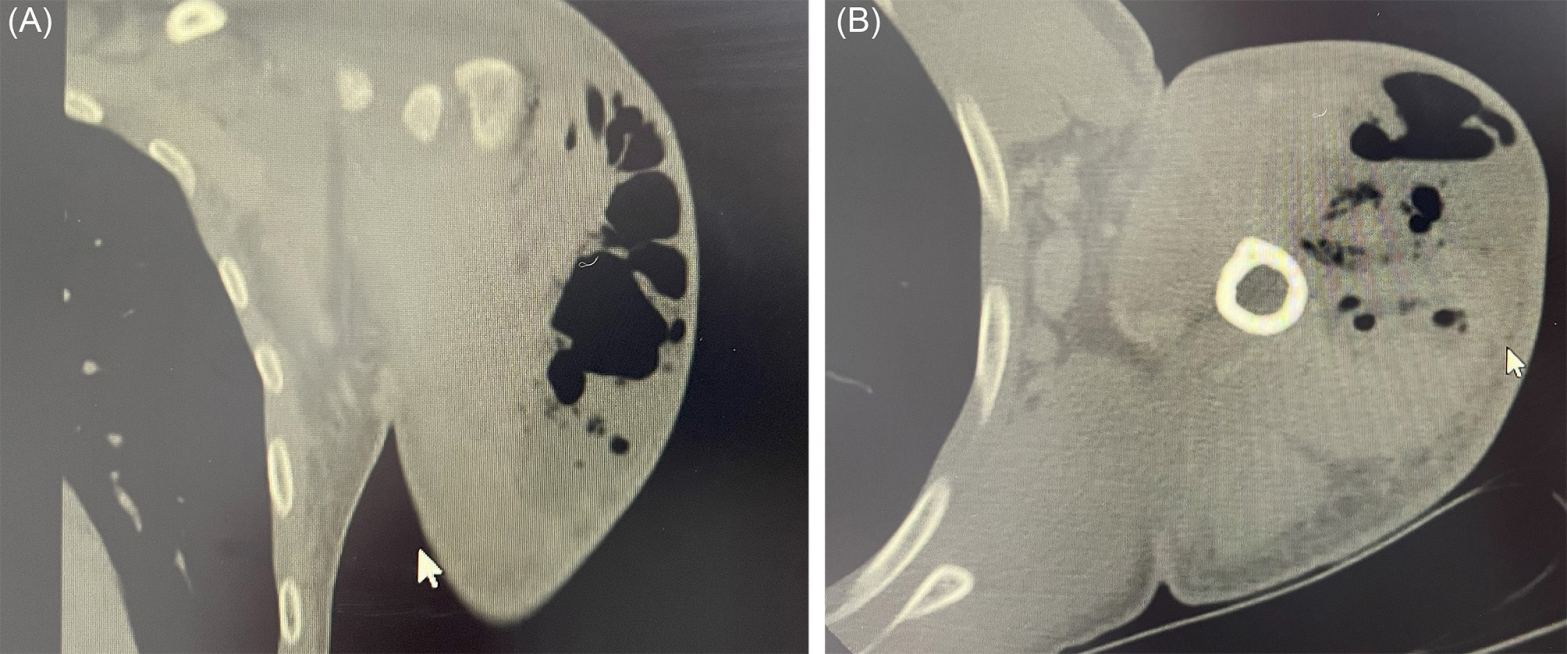
(A) computed coronal tomography: presence of gas. Involvement of rotator cuff tendons, subacromial bursa and acromion; (B) computed tomography with axial analysis: gas in proximal humerus.

We performed a surgical treatment on a 38-year-old patient that had a chronic ketamine use disorder. He came to the Instituto de Infectologia Emilio Ribas, in Sao Paulo State, Brazil, with sepsis due to gas necrosis in his left arm. (Figures 1A and 1B). Even though the patient did not share his needles, he reported inadequate hygiene and storage of needles and syringes, which caused the material contamination that was responsible for the infection in his arm. The patient reported urinary complaints and hallucinations. He had been using a short-term indwelling urethral catheter for two years. The patient underwent tetanus prophylaxis via a booster dose, as he did not have his vaccination records with him.

On the physical examination, he scored 15 points in the Glasgow Coma Scale (GCS) , presenting left arm infection, a respiratory rate of 28 breaths per minute (> 22 breaths per minute), heart rate of 128 beats per minute, blood pressure of 90 × 70 mmHg (systolic pressure < 100 mmHg) and a fever of 39 °C. Laboratory tests showed a 19,500 white blood cell count and 360 mg/ L of C-reactive protein.

**Figure 2:**
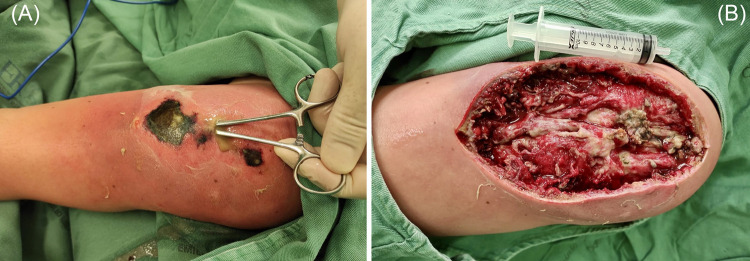
(A) deep infection with necrosis and an abscess; (B) Intra-operatory. Left arm with lesion measuring approximately 18,43 cm x 14,56 cm.

We performed three surgical procedures. Figures 2A and 2B show an extensive lesion involving deep tissues such as muscle and bone.

**Figure 3:**
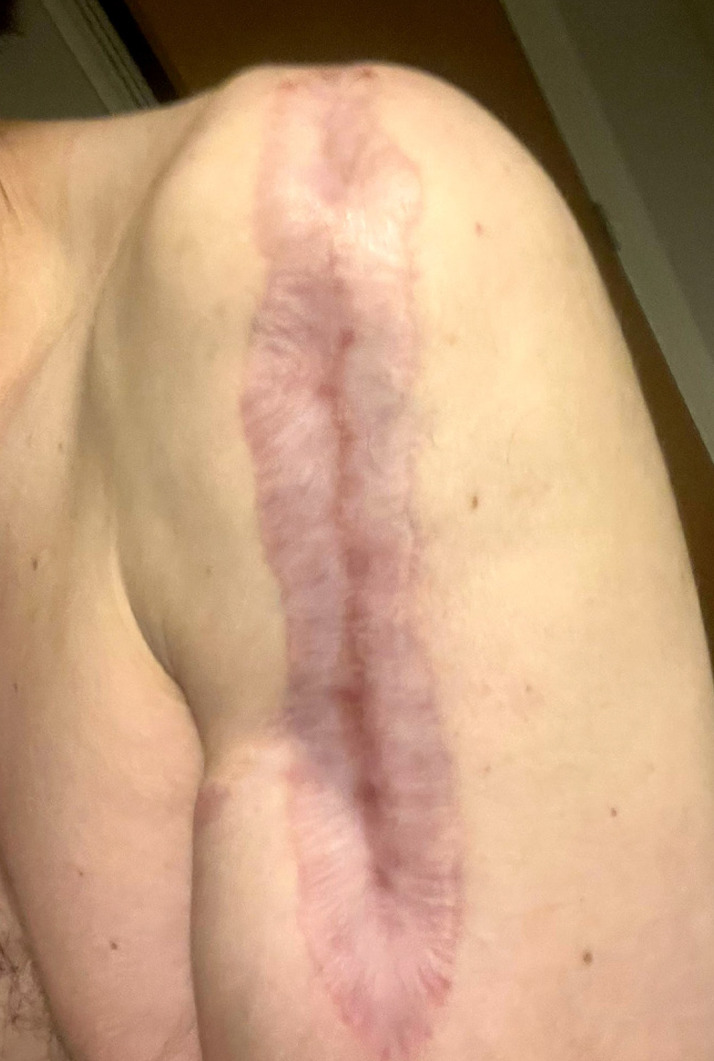
Post surgery. No motor deficits.

Initially, the patient met the criteria for amputation. Due to his age and profession (musician), he chose to try to save the member. Despite numerous surgical procedures, there were no motor and sensory deficits. *Streptococcus acidominimus, Kluyvera ascorbate*, and *Arcnobacterium haemolyticum* were present in the culture. Treatment was performed with vancomycin and meropenem. He remained hospitalized for 46 days and returned to playing the piano within 73 days. Figure 3 shows full healing after 8 months.

## DISCUSSION

Following a multidisciplinary approach, we reinforced accidental tetanus prevention, as injectable drug users are at risk of contracting it^8,9^. Kobayashi *et al*.^10^ reported that the exact mechanism of action underlying “club drug” hepatotoxicity is unknown. Epidemiological ketamine abuse surveys have not clarified the contribution of alcohol to gastrointestinal symptoms. However, reports have highlighted that the co-occurrence of ketamine and alcohol abuse has been prevalent^2,10^. In patients with depression, ketamine restores brain circuits by blocking glutamate (amino acid) action on NMDA receptors (N-methyl-D-aspartate–NMDAR). This effect can last for hours with just one dose. In the reported case, the patient self-administered ketamine three times per day^2,11^.

Contrary to what many healthcare professionals know, ketamine is not FDA approved for treating psychiatric disorders^4,5^. Long-term safety and efficacy of ketamine (esketamine) as an antidepressant requires further study^1,5^. Several studies recommend the use of ketamine for mood disorders, obesity and palliative care. However, therapeutic use often triggers illicit use^1,7^. Unfortunately, inappropriate ketamine use can cause pleiotropic effects, namely: the inhibition of cholinergic transmission, increased systolic pressure, palpitations, nausea, bladder toxicity, euphoria, hallucinations and death^3,5,6,10^. Changes in the neurological system and epithelial repair create bladder toxicity. Chronic ketamine use changes the bladder epithelium and can cause hematuria and urinary obstruction. During treatment, the patient’s urinary routine was normalized^11,12^.

In addition to the resolution of the urological condition, the patient had sepsis regression. On the first day, despite scoring 15 in the GCS, he presented a respiratory rate of 28 breaths per minute (>22 breaths per minute) and blood pressure of 90 mmHg (systolic pressure ≤100 mmHg) with quick Sequential Organ Failure Assessment (qSOFA) ≥2^13-15^. A 2016 SCCM/ESICM task force has defined sepsis as life-threatening organ dysfunction caused by a deregulated host response to infection^16,17^. Since 2016, the terms “severe sepsis” and “systemic inflammatory response syndrome” are no longer used^16,18^. Several clinical and laboratory data points are typically required for the diagnosis of sepsis and septic shock^16,17^.

The two most commonly used scores are the National Early Warning Score (NEWS) and the qSOFA. Both are not organ dysfunction scores, nor are they diagnostic of sepsis. They do not determine individual treatment strategies or predict mortality^15,17,19^.

Today, there are numerous ways of approaching patients with sepsis. Regardless of the scores used, everyone must be quick and know the severity of the condition ^15,18,19^.

Some authors believe that the problem should be solved prior to medical treatment. In Brazil, an ampoule of ketamine costs R$ 90.00 (U$ 16.3). A 50 ml dehydrated ampoule can yield up to 10 grams. Ketamine is sold for R$ 100.00 (U$ 18.1) to R$ 150.00 (U$ 27.2) per gram.

The patient purchased ketamine for veterinary use through the internet without a medical prescription. The scientific community, as well as the police and the government, must ponder the prescription, efficacy and safety of ketamine for diverse medical treatments.

Ketamine use is increasing worldwide due to the ease of access to it. In many countries, toxicological screening already includes a test for ketamine^20^.

## CONCLUSION

Due to the severity of the complications caused by the inappropriate use of legal drugs, a multidisciplinary approach in healthcare is mandatory to minimize after-effects and improve prognosis.

A multidisciplinary approach is also very important for prevention. It must be performed before users reach lethal or irreversible clinical complications. Unfortunately, there are few services available for the treatment of drug addicts in most countries.
